# Macroeconomic changes and educational inequalities in traffic fatalities in the Baltic countries and Finland in 2000–2015: a register-based study

**DOI:** 10.1038/s41598-021-81135-5

**Published:** 2021-01-27

**Authors:** Andrew Stickley, Aleksei Baburin, Domantas Jasilionis, Juris Krumins, Pekka Martikainen, Naoki Kondo, Mall Leinsalu

**Affiliations:** 1 Stockholm Centre for Health and Social Change (SCOHOST), Södertörn University, 141 89 Huddinge, Sweden; 2 Department of Social Epidemiology, Graduate School of Medicine and School of Public Health, Kyoto University, Kyoto, Japan; 3 Department of Epidemiology and Biostatistics, National Institute for Health Development, Tallinn, Estonia; 4 Max Planck Institute for Demographic Research, Rostock, Germany; 5 Demographic Research Centre, Vytautas Magnus University, Kaunas, Lithuania; 6 Demography Unit, Faculty of Business, Management and Economics, University of Latvia, Riga, Latvia; 7 Population Research Unit, University of Helsinki, Helsinki, Finland; 8 Centre for Health Equity Studies (CHESS), Stockholm University and Karolinska Institutet, Stockholm, Sweden; 9 Institute for Future Initiatives, The University of Tokyo, Tokyo, Japan

**Keywords:** Medical research, Epidemiology

## Abstract

This study examined trends and inequalities in road traffic accident (RTA) mortality in the Baltic countries (Estonia, Latvia, Lithuania) and Finland in relation to large-scale macroeconomic changes in the 2000s. Educational inequalities in RTA mortality in 2000–2003, 2004–2007, 2008–2011 and 2012–2015 among 30–74 year olds were examined using census-linked longitudinal mortality data and by estimating the relative and slope index of inequality. Overall RTA mortality decreased substantially between 2000–2003 and 2012–2015. From 2004–2007 to 2008–2011, the RTA mortality decline accelerated but was larger in the Baltic countries. Among men the RTA mortality decline was mostly driven by a larger fall among the high and middle educated. Among women, the changes in RTA mortality by educational level had no clear pattern. From 2000–2003 to 2012–2015 relative educational inequalities in RTA mortality increased among men, although more in the Baltic countries. Among women the pattern was mixed across countries. Absolute inequalities fell in all countries among both sexes. Educational inequalities in male RTA mortality may be growing because of increasingly less access to safer cars and a more hazardous driving culture among the lower educated.

## Introduction

It has been estimated that globally, there were 1.35 million road traffic deaths in 2016^1^, while between 20 and 50 million people suffered non-fatal injuries, many of which will have resulted in disability^2^. Between 2013 and 2016 the number of deaths from road traffic accidents (RTA) increased in 104 countries across the world, with road traffic injuries now comprising the eighth leading cause of death for all age groups worldwide^1^. Besides the severe toll that RTA exert on public health and wellbeing, they also have a detrimental economic impact with a recent study estimating that road injuries would cost the world economy as much as 1.8 trillion US$ in the period between 2015 and 2030 (constant at 2010 US$)^3^.

Understanding what factors are associated with RTA mortality thus has a clear public health imperative, especially as a recent report from the World Health Organisation (WHO) has indicated that it is unlikely given recent trends, that the United Nations Sustainable Development Goals (SDG) subtarget 3.6—to halve road traffic deaths and injuries by 2020—will be met^1^. In connection with this, the current study will examine the association between large-scale economic changes and social inequalities in RTA mortality. Several previous studies have looked at the effects of both short- and long-term economic change on RTA mortality. Studies using data from the United States in 1976–2010^4^, and 1988–2010^5^, and the Organisation for Economic Co-operation and Development (OECD) countries in 1970–2010^6^, have all reported that RTA mortality is pro-cyclical. Other research has focused on the effects of the Great Recession (2008–2010) with studies from both Spain^7^ and Great Britain^8^ showing that it was associated with a reduction in RTA mortality, while another study indicated that in OECD countries it may have also been linked to an accelerated decline in mortality over and above prior trends^9^.

Despite these general findings, as yet, relatively little is known about whether the effects of economic change on RTA mortality differ by socioeconomic status. This may be an important omission. Previous research has indicated that there is an educational gradient in RTA outcomes with lower education being associated with an increased risk of both injury and death^10,11^. Moreover, research from the United States has suggested that socioeconomic inequality in RTA mortality may have widened in recent years as a result of more favourable mortality trends in more highly educated groups^12^. It is uncertain what role economic fluctuations might have played in such changes although a recent study from Argentina that focused on educational inequalities in RTA mortality in 1999–2013, found that inequalities decreased in the period of economic crisis, while economic expansion was associated with RTA mortality increasing only among the low educated^13^. Mirroring this, other research from Great Britain showed that the least affluent groups may have experienced a slightly larger reduction in RTA mortality in the Great Recession in 2008–2010 vs. 2004–2007^8^.

The current study will use data from the Baltic countries—Estonia, Latvia, Lithuania—and Finland in 2000–2015. These countries may offer an informative setting to examine the effects of economic change on educational inequalities in RTA mortality. Specifically, they underwent large-scale macroeconomic changes in the 2000s: between 2000 and 2008 per capita gross domestic product (GDP) quadrupled in the Baltic countries, while in Finland it more than doubled during the same years. In all countries, growth accelerated in the second half of this period. However, this growth halted abruptly with the onset of the global financial crisis which had a deeply negative impact on the Baltic countries where per capita GDP fell sharply in 2008 to 2009 (> 20% on average), while in Finland it decreased by about 12%. The speed of economic recovery also varied across the countries: by 2013 Estonia and Lithuania had surpassed their pre-recession levels in terms of per capita GDP, while in Latvia and Finland, even by as late as 2015, GDP remained below its 2008 level^14^.

Changes in national wealth are likely to impact the rate of motorization and may be associated with traffic fatalities. In 2015 there were 590 passenger cars per 1000 inhabitants in Finland—a 43% increase from 2000. The respective numbers in Estonia were 515 (a 56% increase), 343 in Latvia (a 46% increase) and 428 in Lithuania (a 28% increase)^15^. Passenger cars predominated and comprised approximately 80% to 90% of the road vehicle fleet in our study countries in both 2000 and 2015^15^. Traffic fatalities are also related to national road safety policies. These were similar across the study countries. For example, the speed limit on urban roads was 50 km/h across the four countries, while similar motorcycle helmet and seat belt laws were also in place^16^. At the same time, the blood alcohol concentration (BAC) limits for the general population were somewhat lower in Estonia (0.02 g/dl), than in Lithuania (0.04 g/dl) and Latvia and Finland (0.05 g/dl)^16^, while there is also some indication that the enforcement of these policies may have been slightly weaker in Lithuania than in our other study countries^16^. Despite relatively strict drink driving regulations alcohol may play a crucial role in traffic fatalities in the Baltic countries and Finland because of high overall consumption levels and somewhat risky drinking patterns^17^. In terms of the current study this might be important as alcohol consumption has also been related to macroeconomic change^18^.

Thus, this study aimed to examine the trends and educational inequalities in mortality from RTA between 2000 and 2015 in the Baltic countries and Finland in the context of large-scale macroeconomic changes.

## Methods

### Data

Data for Estonia, Latvia and Lithuania originate from longitudinal mortality follow-up studies of population censuses in 2000 (2001 in Lithuania) and 2011 where all permanent residents who participated in the census were followed from the census date until the date of death or emigration, or until the end of the follow-up period, i.e. either 31.12.2011 (for the 2000/2001 census) or 31.12.2015 (2011 census). The date and cause of death were linked from national mortality registries with 95–98% of deaths being successfully matched to census records. All data linkages were performed by National Statistical Offices. Data for Finland were obtained from the longitudinal register-based population data file of Statistics Finland covering the total population. Data were organised into four sub-periods representing moderate economic growth (2000–2003), economic expansion (2004–2007), recession (2008–2011), and a stabilisation period (2012–2015). The population exposures for those aged 30 years and older were calculated by adding up the number of person years lived by each individual within each 5-year age interval in a given period. Deaths were allocated to age groups using the age at death. Anonymised data were aggregated into multidimensional frequency tables combining deaths and population exposures split by study periods and sociodemographic variables before they were delivered for research purposes. This study included persons aged 30–74 years. This age cut-off was used to ensure that the full educational history of the subjects was likely to have been recorded.

RTA deaths were classified as V01–V89, Y85 according to the 10th revision of the International Classification of Diseases (ICD-10). Sociodemographic characteristics are census based and were coded by Statistical Offices following a common study protocol. Educational level was categorised as *low,* referring to primary and lower secondary education (International Standard Classification of Education 2011^19^ categories 0–2), *middle,* including upper secondary and post-secondary non-tertiary education (3–4), and *high,* covering tertiary education (5–8). The percent of missing values for education was low (0–0.8%) and these cases were omitted from the analysis that included 12,621 RTA deaths and about 104 million person years (Table 1).

**Table 1:** Characteristics of the study populations in 2000–2015 among men and women in the 30–74 age group.

Country	Period	Men						Women					
		Deaths	Person years	Educational level (% of person years)				Deaths	Person years	Educational level (% of person years)			
		N	N	High	Middle	Low	Missing	N	N	High	Middle	Low	Missing
Finland	2000–2003	781	5,792,113	26.6	37.5	35.9	0.0	278	5,929,293	30.3	34.9	34.8	0.0
	2004–2007	659	5,805,170	27.0	41.0	32.0	0.0	229	5,904,788	32.4	37.5	30.1	0.0
	2008–2011	527	5,857,096	25.8	45.2	29.0	0.0	169	5,941,203	32.1	41.5	26.4	0.0
	2012–2015	484	6,106,322	31.4	43.7	24.9	0.0	143	6,169,810	40.8	38.7	20.5	0.0
Estonia	2000–2003	361	1,233,736	25.5	47.4	27.1	0.7	96	1,530,689	34.1	43.8	22.1	0.5
	2004–2007	336	1,306,910	25.6	50.5	23.9	0.7	108	1,607,904	35.6	46.5	17.9	0.5
	2008–2011	195	1,300,849	24.5	53.1	22.4	0.8	75	1,578,775	35.4	49.6	15.0	0.5
	2012–2015	122	1,333,700	31.0	49.8	19.2	0.3	58	1,553,757	45.0	43.1	11.9	0.1
Latvia	2000–2003	916	1,946,570	15.4	57.1	27.5	0.6	281	2,487,960	18.6	57.3	24.1	0.4
	2004–2007	792	2,042,929	15.4	59.9	24.7	0.6	250	2,585,262	19.6	60.4	20.0	0.4
	2008–2011	422	2,023,853	14.7	61.5	23.8	0.4	169	2,523,695	19.4	63.5	17.1	0.3
	2012–2015	329	1,903,683	22.2	63.6	14.2	0.1	104	2,330,657	33.4	56.6	10.0	0.0
Lithuania	2001–2003	887	2,426,813	16.4	59.0	24.6	0.5	278	2,949,919	18.8	57.9	23.3	0.4
	2004–2007	1442	3,409,556	16.4	60.9	22.7	0.5	476	4,068,614	19.4	60.8	19.8	0.5
	2008–2011	726	3,390,429	16.2	62.5	21.3	0.5	251	4,015,691	19.7	64.0	16.3	0.5
	2012–2015	498	3,072,742	22.8	59.8	17.4	0.0	179	3,661,689	30.3	56.9	12.8	0.0

The follow up in the 1st period started from the census date in the Baltic countries, i.e. 31.03.2000 in Estonia, 1.03.2000 in Latvia, and 6.04.2001 in Lithuania, on all other occasions the follow up started on January 1 and ended on December 31 in the respective periods.

### Statistical analysis

Age-standardised mortality rates (ASMRs) per 100,000 person years were calculated for total RTA mortality and separately by educational level using the European Standard Population^20^. Changes in RTA mortality between two consecutive periods were assessed using age-adjusted mortality rate ratios (RRs) calculated using Poisson regression, with the preceding period as the reference category (RR = 1). The results from the Poisson regression analysis were verified by using a negative binomial regression analysis that produced similar effect sizes (the results are available from the authors upon request). Educational inequalities in RTA mortality were assessed using the relative index of inequality (RII) and slope index of inequality (SII)^21^. The RII and SII are regression-based measures that adjust the relative position of each educational group to its share in the population. The relative position is determined with educational rank, a cumulative proportion of each educational group within the educational hierarchy, with 0 (for the highest educated) and 1 (for the lowest educated) as the extreme values on the rank order. RIIs were calculated with Poisson regression and were adjusted for 5-year age groups. The RII can be interpreted as the mortality rate ratio comparing those with the lowest educational level to those with the highest educational level. The SII measures absolute mortality rate differences between the lowest and highest end of the educational hierarchy. SIIs were calculated from the RIIs and the overall ASMRs by using the formula SII = 2*ASMR*(RII − 1)/(RII + 1). The ASMRs and RIIs were calculated using SPSS Statistics for Windows, version 26.0 (IBM Corp. 2019) and RRs were calculated using STATA 14.2 (Stata Corp., College Station, Texas, USA).

## Results

**Figure 1:**
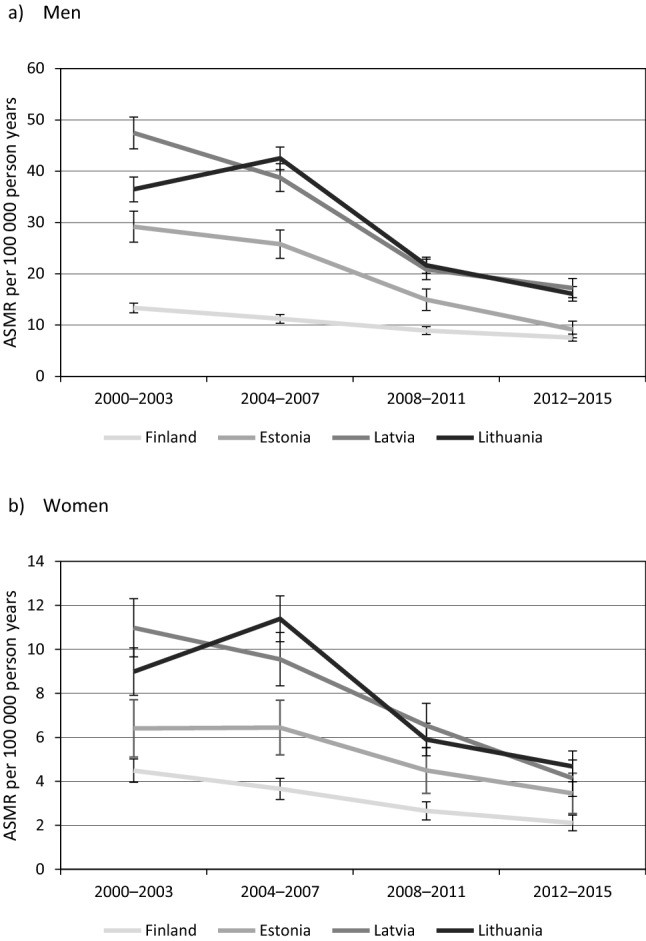
Age-standardised mortality rate (ASMR) per 100,000 person years with 95% CIs for road traffic accidents.

**Figure 2:**
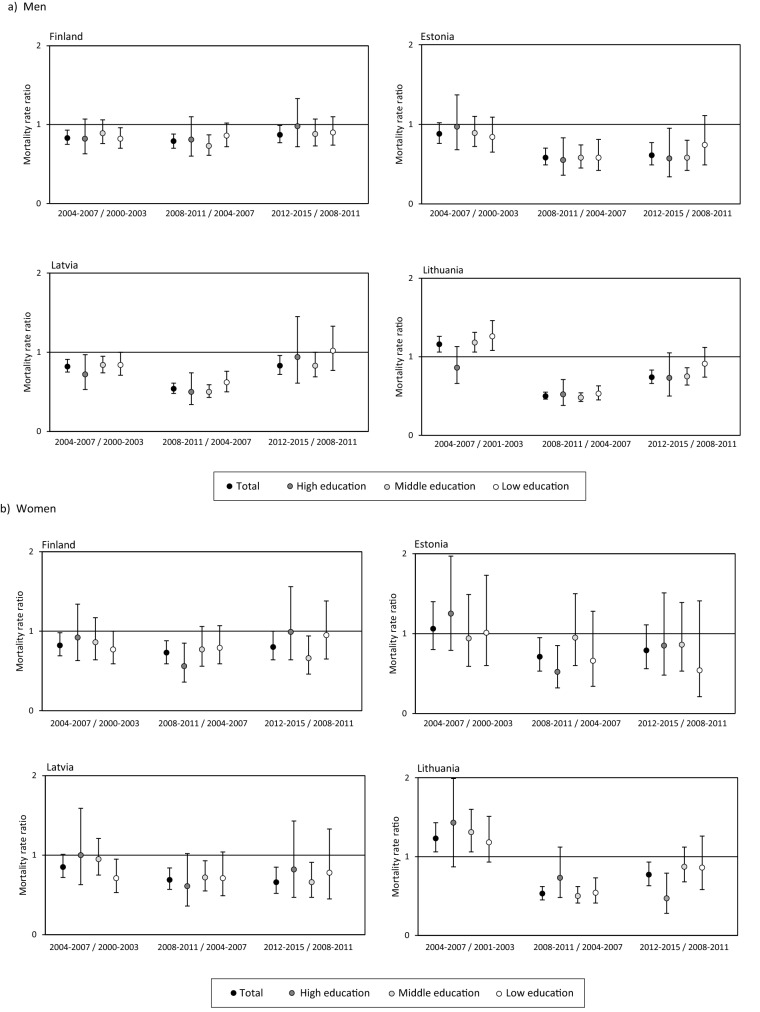
Change in road traffic accident mortality between two consecutive periods assessed by age-adjusted mortality rate ratios (preceding period = 1) with 95% CIs.

The ASMRs per 100,000 person years for overall RTA mortality ranged from 13.4 to 47.5 among men and from 4.5 to 11.0 among women in 2000–2003 and from 7.6 to 17.2 among men and from 2.1 to 4.7 among women in 2012–2015 (Tables 2 and 3). Although the ASMRs were consistently higher in the Baltic countries than in Finland the gap narrowed substantially by 2012–2015 (Fig. 1). As assessed by mortality rate ratios (Fig. 2; Supplementary Table 1), RTA mortality increased in Lithuania, but decreased in Finland and among men in Latvia between 2000–2003 and 2004–2007. From 2004–2007 to 2008–2011 the RTA mortality decline accelerated among both men and women in all countries, with a larger fall observed in the Baltic countries. Among men, RTA mortality continued to decline from 2008–2011 to 2012–2015, although at a slower pace; among women it declined only in Latvia and Lithuania.

**Table 2:** Changes and educational inequalities in road traffic accident mortality in 2000–2015 among men in the 30–74 age group.

Country	Period	Total	Educational level			RII (95% CI)	SII
			High	Middle	Low		
		ASMR (95% CI)	ASMR (95% CI)	ASMR (95% CI)	ASMR (95% CI)		
Finland	2000–2003	13.4 (12.4–14.3)	7.6 (6.2–9.0)	14.0 (12.1–15.8)	17.6 (15.6–19.5)	2.92 (2.19–3.88)	13.1
	2004–2007	11.2 (10.4–12.1)	6.2 (4.9–7.5)	12.0 (10.5–13.5)	15.3 (13.3–17.3)	2.97 (2.19–4.01)	11.1
	2008–2011	8.9 (8.2–9.7)	5.2 (3.8–6.5)	8.7 (7.6–9.9)	14.2 (12.1–16.3)	3.47 (2.48–4.86)	9.8
	2012–2015	7.6 (6.9–8.2)	4.6 (3.7–5.6)	7.7 (6.6–8.7)	11.6 (9.7–13.5)	3.12 (2.21–4.40)	7.8
Estonia	2000–2003	29.2 (26.2–32.2)	19.9 (15.0–24.9)	27.9 (23.4–32.3)	43.1 (34.6–51.6)	2.81 (1.86–4.26)	27.8
	2004–2007	25.8 (23.0–28.5)	19.4 (14.6–24.1)	25.2 (21.2–29.2)	35.5 (27.8–43.1)	2.12 (1.39–3.23)	18.5
	2008–2011	15.0 (12.9–17.1)	10.7 (7.0–14.4)	15.5 (12.4–18.6)	21.0 (15.0–27.0)	2.15 (1.24–3.73)	11.0
	2012–2015	9.2 (7.5–10.8)	6.2 (3.8–8.6)	8.9 (6.6–11.1)	17.4 (11.3–23.5)	3.46 (1.71–6.98)	10.1
Latvia	2000–2003	47.5 (44.4–50.5)	34.3 (27.5–41.0)	44.6 (40.6–48.6)	64.8 (56.5–73.1)	2.20 (1.68–2.88)	35.6
	2004–2007	38.8 (36.0–41.5)	24.1 (18.7–29.6)	37.7 (34.2–41.2)	52.9 (45.4–60.3)	2.34 (1.75–3.11)	31.1
	2008–2011	20.9 (18.9–22.9)	12.6 (8.3–16.9)	19.0 (16.6–21.5)	32.0 (26.1–37.8)	3.33 (2.25–4.94)	22.5
	2012–2015	17.2 (15.4–19.1)	11.5 (8.3–14.7)	15.6 (13.4–17.8)	29.5 (22.6–36.4)	3.86 (2.43–6.15)	20.3
Lithuania	2001–2003	36.5 (34.0–38.9)	23.7 (18.9–28.5)	33.6 (30.4–36.9)	49.3 (42.3–56.4)	2.62 (1.97–3.50)	32.7
	2004–2007	42.5 (40.3–44.7)	20.3 (16.6–24.0)	41.7 (38.7–44.6)	63.5 (56.5–70.5)	3.50 (2.80–4.37)	47.2
	2008–2011	21.7 (20.1–23.3)	10.5 (7.8–13.2)	20.9 (18.8–22.9)	34.0 (28.7–39.2)	3.58 (2.62–4.89)	24.4
	2012–2015	16.1 (14.7–17.5)	7.8 (5.7–9.9)	15.5 (13.6–17.3)	29.8 (24.5–35.1)	5.35 (3.68–7.76)	22.1

*ASMR* age-standardised mortality rate per 100,000 person years, *CI* confidence interval, *RII* relative index of inequality, *SII* slope index of inequality per 100,000 person years.

**Table 3:** Changes and educational inequalities in road traffic accident mortality in 2000–2015 among women in the 30–74 age group.

Country	Period	Total	Educational level			RII (95% CI)	SII
			High	Middle	Low		
		ASMR (95% CI)	ASMR (95% CI)	ASMR (95% CI)	ASMR (95% CI)		
Finland	2000–2003	4.5 (4.0–5.0)	3.3 (2.4–4.3)	4.5 (3.5–5.5)	6.1 (4.7–7.4)	2.02 (1.23–3.31)	3.0
	2004–2007	3.7 (3.2–4.1)	3.1 (2.3–4.0)	3.9 (3.1–4.8)	4.4 (3.2–5.6)	1.44 (0.86–2.41)	1.3
	2008–2011	2.7 (2.2–3.1)	1.8 (1.2–2.4)	3.0 (2.3–3.7)	3.7 (2.5–4.9)	2.22 (1.22–4.04)	2.0
	2012–2015	2.1 (1.7–2.5)	1.7 (1.2–2.3)	2.0 (1.4–2.5)	3.6 (2.2–5.0)	2.05 (1.08–3.89)	1.4
Estonia	2000–2003	6.4 (5.1–7.7)	6.5 (4.2–8.7)	5.2 (3.5–6.9)	12.6 (6.2–19.0)	1.61 (0.72–3.58)	3.0
	2004–2007	6.4 (5.2–7.7)	8.0 (5.6–10.3)	4.9 (3.3–6.5)	10.9 (5.1–16.7)	0.76 (0.37–1.56)	− 1.8
	2008–2011	4.5 (3.5–5.5)	3.7 (2.2–5.2)	4.8 (3.2–6.3)	8.2 (2.6–13.9)	1.54 (0.64–3.67)	1.9
	2012–2015	3.5 (2.5–4.4)	3.2 (1.9–4.5)	3.9 (2.4–5.4)	3.4 (0.2–6.5)	1.02 (0.38–2.72)	0.1
Latvia	2000–2003	11.0 (9.7–12.3)	7.3 (4.8–9.7)	9.4 (7.8–11.0)	25.5 (18.6–32.3)	3.92 (2.34–6.55)	13.1
	2004–2007	9.6 (8.3–10.8)	7.5 (5.1–9.9)	8.9 (7.4–10.3)	16.7 (11.7–21.7)	2.34 (1.38–3.97)	7.7
	2008–2011	6.5 (5.5–7.5)	4.5 (2.5–6.4)	6.3 (5.1–7.5)	12.8 (7.8–17.7)	2.76 (1.45–5.26)	6.1
	2012–2015	4.1 (3.3–5.0)	3.6 (2.3–5.0)	3.9 (2.9–5.0)	9.3 (3.9–14.8)	1.88 (0.85–4.16)	2.5
Lithuania	2001–2003	9.0 (7.9–10.1)	4.3 (2.5–6.0)	8.5 (7.0–9.9)	16.8 (10.9–22.7)	4.42 (2.51–7.76)	11.4
	2004–2007	11.4 (10.3–12.4)	6.3 (4.5–8.1)	11.0 (9.7–12.4)	23.8 (18.0–29.6)	4.24 (2.79–6.44)	14.1
	2008–2011	5.9 (5.2–6.6)	4.4 (3.0–5.8)	5.7 (4.8–6.6)	11.5 (7.4–15.6)	2.13 (1.26–3.62)	4.3
	2012–2015	4.7 (4.0–5.4)	2.1 (1.3–3.0)	5.0 (4.0–6.0)	10.5 (6.7–14.4)	6.31 (3.29–12.09)	6.8

*ASMR* age-standardised mortality rate per 100,000 person years, *CI* confidence interval, *RII* relative index of inequality, *SII* slope index of inequality per 100,000 person years.

From 2000–2003 to 2004–2007 RTA mortality decreased among high and middle educated men in Latvia, among low educated men in Finland, but increased among middle and low educated men in Lithuania (Fig. 2; Supplementary Table 1). Over the same time period, RTA mortality decreased among low educated women in Latvia but increased among middle educated women in Lithuania. Between 2004–2007 and 2008–2011, the RTA mortality decline among men was larger among high and/or middle educated in all countries; among women the decline was larger among high educated in Finland and Estonia but among middle and low educated in Lithuania and it also declined among middle educated women in Latvia. From 2008–2011 to 2012–2015, RTA mortality continued to decline among high and middle educated men in Estonia and among middle educated men in Lithuania. Among women it only declined among the high educated in Lithuania and among middle educated in Finland and in Latvia.

Relative educational inequalities (RIIs) in RTA mortality among men ranged from 2.20 in Latvia to 2.92 in Finland in 2000–2003 (Table 2). Between 2000–2003 and 2012–2015 the RIIs increased in all countries, however, the increase was larger in the Baltic countries. Absolute inequalities (SIIs) declined over the study period in all countries but remained higher in Latvia and Lithuania. Among women (Table 3), sizeable relative inequalities in RTA mortality in 2000–2003 were observed in Latvia (RII = 3.92) and Lithuania (RII = 4.42) but were somewhat lower in Finland (RII = 2.02). By 2012–2015, the RII was substantially reduced in Latvia, whereas the RII had increased in Lithuania and remained the same in Finland. Absolute inequalities in RTA mortality among women decreased over the study period in all countries.

## Discussion

There was a substantial decrease in overall RTA mortality between 2000–2003 and 2012–2015. From 2004–2007 to 2008–2011 the decline in RTA mortality accelerated in all countries, but was larger in the Baltic countries than in Finland which resulted in a narrowing RTA mortality gap by 2012–2015. Among men the RTA mortality decline was mostly underpinned by a larger fall among the high and middle educated. Among women, there was no clear pattern to the changes in RTA mortality by educational level. From 2000–2003 to 2012–2015 relative educational inequalities in RTA mortality increased among men in all countries, although the increase was larger in the Baltic countries. Among women relative inequalities increased only in Lithuania, while inequalities were not observed or were reduced in Estonia and Latvia. Absolute inequalities fell in all countries among both sexes.

The major strength of this study is that it uses longitudinal register-based data and is specifically designed to examine inequalities in RTA mortality in relation to economic fluctuations over a 15-year period in the Baltic countries and Finland, thus achieving the best possible comparability across countries and over time. However, the study also has some limitations that should be considered before discussing the key findings. The censuses in the Baltic countries combined traditional population survey-based enumeration, electronic self-enumeration (in 2011), and register-based enumeration. The share of the population enumerated using only survey-based and electronic enumeration varied from 91% in Latvia to 98% in Estonia^22^. The register-based data did not cover information about educational level and were thus excluded from the main analysis. To assess the magnitude and direction of the potential bias we performed a sensitivity analysis for Latvia comparing mortality estimates while excluding and including register-based data. By excluding register-based records we slightly underestimated overall RTA mortality but the effect on changes between the periods was minimal (Supplementary Table 2). Although we cannot exclude the possibility that the effect differed by educational level, we believe that this bias would have had only a marginal impact on our conclusions related to the changes in RTA mortality in Latvia. Another limitation is that we were not able to examine RTA mortality for different road users and transport modes. This is an important omission given that there may be variations between the countries in terms of the prevalence of different forms of transport deaths (e.g. pedestrians, car occupants, motorcyclists)^23–25^ that might also be linked to differences in educational attainment. Future research should examine the role of socioeconomic inequalities in different forms of transport mortality. Finally, although we studied changes in RTA mortality in the context of macroeconomic changes, macroeconomic variation was determined only by study period and, thus, we cannot rule out the possibility of confounding due to other temporal changes.

Much of the previous research on the association between economic change and RTA mortality has highlighted the pro-cyclical nature of this association^4–6^. A recent study that examined the short- and long-term effects of GDP on traffic deaths in 18 OECD countries in 1960–2011 found that although cyclical periods of strong economic growth led to an immediate increase in traffic deaths, since the mid-1970s this short-term effect has been outweighed by a much stronger protective long-term effect with a decreasing trend observed in death rates^26^. The results of our study largely accord with this finding as apart from Lithuania in 2004–2007, RTA mortality declined among adults in all countries across the period with a larger reduction observed in the Baltic countries. The accelerated decline observed in 2008–2011—especially in the Baltic countries—is also in line with the finding of Wegman and colleagues that the more severe a recession is, the steeper the decline in traffic deaths^9^.

It is possible that different mechanisms might be associated with reduced RTA mortality in different economic phases and time periods. For instance, it is likely that over longer periods of economic growth increases in wealth bring about a greater number of newer vehicles with improved safety features on the roads reducing both the risk of RTAs and RTA fatalities^27^. Indeed, car sales grew sharply in the Baltic countries in the years of economic expansion. Specifically, between 2004 and 2007 the registration of new passenger cars grew by 1.9 times in Estonia and Latvia and 1.6 times in Lithuania^15^. Similarly, economic expansion may allow greater investment in transport infrastructure to create a safer driving environment. In particular, European Union (EU) structural funding, which was used to help improve road infrastructure in the years following EU accession^28^, may have played an important role in improving road safety in the Baltic countries. For example, investment in road transport infrastructure in Estonia grew from 56 to 142 million Euros in 2004–2008^29^, which among other things, resulted in an improvement in the condition of trunk roads with the average age of the surfacing starting to decline^30^. Alternatively, in times of recession it is possible that factors such as a reduced volume of traffic, including less private car use^31^, and a change in driving behaviour, such as reduced driving speed in order to improve the fuel efficiency of vehicles^32^ when real fuel prices may be increasing^33^, might be important in this context. There is also some evidence that recession may be associated with a reduction in overall alcohol consumption and drinking and driving possibly as a consequence of reduced expenditure^34^. In particular, there was a sharp reduction in alcohol consumption between 2007 and 2009 in Estonia from 14.8 L per capita to 11.9 among those aged 15 and above^35^ and in Latvia (12.1 to 9.9) while there was little change in Finland (10.5 to 10.0)^36^; after that consumption levels did not reach their pre-recession levels again^35,36^. These changes seem to be mirrored in RTA mortality involving alcohol. Specifically, while 48% of all RTA deaths involved alcohol in Estonia in 2007 by 2013 this figure had fallen to 25% with comparable figures for Latvia being 21% (2006) and 6%. At the same time, in Finland, the share of alcohol-related RTA deaths only dropped from 24 to 22%^16,37^.

It is uncertain why RTA mortality increased in Lithuania in 2004–2007 although several factors may have been involved. In particular, there is some evidence that alcohol was associated with RTA mortality in Lithuania throughout the late 1990s to 2007^38^. Similar to other Baltic countries, alcohol consumption dropped in Lithuania from 2007 (13.8 L per capita) to 2009 (13.0)^36^, probably as a result of the combined effect of increased excise tax and declining income^39,40^, thus contributing to accelerated RTA mortality decline in 2008–2011. However, the consumption level increased in Lithuania thereafter^36^ and RTA deaths involving alcohol increased from 12–16% between 2006 and 2013^16,37^. As overall RTA mortality continued to fall in 2012–2015 among Lithuanian men this suggests that alcohol is only one, albeit important, factor affecting RTA mortality. This is confirmed by a more recent study from Lithuania showing that comprehensive alcohol control measures (e.g. increases in excise tax in combination with other specific measures such as a reduction in the availability of alcohol) have resulted in notable progress being made in reducing alcohol-related RTA mortality, particularly from 2014 onwards^40^.

In terms of inequalities in RTA mortality, although absolute inequalities reduced among men and women in all countries across the study period, relative inequalities increased among men in every country while the pattern was more varied among women. It can be speculated that several mechanisms might be associated with increasing relative inequality in RTA mortality. For instance, there is some evidence more generally that lower income groups may have less access to safer vehicles^41^. This might have been especially important (for both male and female inequalities in RTA mortality) in a country such as Lithuania, where the trauma care system has been described as being inadequate in terms of meeting the population’s needs^42^. It is also possible that alcohol might have played a role. Studies have reported that alcohol was strongly linked to RTA mortality in the Baltic countries during the study period^38,43^ among both drivers and pedestrians^43^, while other research has shown that a pronounced socioeconomic gradient exists in alcohol consumption in these countries with lower status groups more likely to engage in risky drinking^44^. Importantly, inequalities in alcohol-related mortality have increased in the Baltic countries and in Finland in recent years because of the larger rise in mortality in lower socioeconomic groups^45^ suggesting that alcohol may have an important role in socioeconomic differences in RTA mortality. Although relative inequalities in RTA mortality became more pronounced among men in 2000–2015 in all of the countries, in Estonia there were no relative educational inequalities in RTA mortality among women at any point during the study period, while the noticeable educational gradient that existed among Latvian women had been attenuated by 2015. It is uncertain what underlies these findings and whether they might be associated with the degree of involvement with driving, or educational differences in improved driving behaviours, such as the increased use of front seatbelts that was noted among Latvian women in an earlier study^46^. Future studies should examine driving behaviours and associated factors among different educational groups in these countries to help elucidate these different patterns of educational inequality in RTA mortality between men and women within and between countries.

In conclusion, this study suggests that in Estonia, Latvia and Finland economic growth may have contributed to falling RTA mortality. Although the mechanisms that underpin this change are uncertain, it is possible that larger investments in traffic safety as a result of EU structural funding and access to better cars may have been important. Economic contraction was associated with accelerated RTA mortality decline across all of the countries, possibly via reduced traffic density caused by increased fuel prices and a loss of or a reduction in income affecting driving (distance/behaviours) with a larger effect seen in the less wealthy countries. Across the study period, lower educated men became more disadvantaged in terms of RTA mortality, possibly because of reduced access to safer cars and because of a more harmful drinking and driving culture. Continued investment in road transport infrastructure is important for lowering overall RTA mortality in these countries further. In addition, efforts to expand road user education and training in schools^24,25^ in conjunction with continuing public health campaigns aimed at behaviours such as drinking and driving may also help to reduce inequalities in RTA mortality in these countries.

## Supplementary Information

Supplementary Information.

## Data Availability

Due to the data protection regulations of the respective National Statistical Offices we are not allowed to make the data available to third parties. Interested researchers have the possibility to obtain data by contacting National Statistical Offices directly (Statistics Estonia email: stat@stat.ee; Statistics Lithuania email: statistika@stat.gov.lt; the Central Statistical Bureau of Latvia email: pasts@csb.gov.lv; Statistics Finland email: tutkijapalvelut@stat.fi). Alternatively, contact the authors from the respective countries to enquire about potential research collaboration.
