# Supplementary material for: Macroeconomic changes and educational inequalities in traffic fatalities in the Baltic countries and Finland in 2000–2015: a register-based study
Source: Sci Rep. 2021 Jan 27;11:2397. doi: 10.1038/s41598-021-81135-5 (PMC7840907; doi:10.1038/s41598-021-81135-5)
Supplement: Supplementary file 1 — Supplementary Information. [file 41598_2021_81135_MOESM1_ESM.pdf]

Supplementary Information

Macroeconomic changes and educational inequalities in traffic fatalities in the Baltic countries and Finland in 2000–2015: a register-based study

Andrew Stickley, Aleksei Baburin, Domantas Jasilionis, Juris Krumins, Pekka Martikainen, Naoki Kondo, Mall Leinsalu

Supplementary Table 1. Changes in road traffic accident mortality compared to the preceding period by educational level

Sex	Country	Comparing periods		Educational level		
			Total	High	Middle	Low
			RR (95% CI)	RR (95% CI)	RR (95% CI)	RR (95% CI)
Men	Finland	2004–2007 / 2000–2003	0.83 (0.75–0.93)	0.82 (0.63–1.07)	0.89 (0.76–1.06)	0.82 (0.70–0.96)
		2008–2011 / 2004–2007	0.79 (0.70–0.88)	0.81 (0.60–1.10)	0.73 (0.61–0.87)	0.86 (0.72–1.02)
		2012–2015 / 2008–2011	0.87 (0.77–0.99)	0.98 (0.72–1.33)	0.88 (0.73–1.07)	0.90 (0.74–1.10)
	Estonia	2004–2007 / 2000–2003	0.88 (0.76–1.02)	0.97 (0.68–1.37)	0.89 (0.72–1.10)	0.84 (0.65–1.09)
		2008–2011 / 2004–2007	0.58 (0.49–0.70)	0.55 (0.36–0.83)	0.58 (0.45–0.74)	0.58 (0.42–0.81)
		2012–2015 / 2008–2011	0.61 (0.49–0.77)	0.57 (0.34–0.95)	0.58 (0.42–0.80)	0.74 (0.49–1.11)
	Latvia	2004–2007 / 2000–2003	0.82 (0.75–0.91)	0.72 (0.53–0.97)	0.84 (0.74–0.95)	0.84 (0.71–1.00)
		2008–2011 / 2004–2007	0.54 (0.48–0.61)	0.50 (0.34–0.74)	0.50 (0.43–0.59)	0.62 (0.50–0.76)
		2012–2015 / 2008–2011	0.83 (0.72–0.96)	0.94 (0.61–1.45)	0.83 (0.69–1.00)	1.02 (0.77–1.33)
	Lithuania	2004–2007 / 2000–2003	1.16 (1.06–1.26)	0.86 (0.66–1.13)	1.18 (1.06–1.31)	1.26 (1.08–1.46)
		2008–2011 / 2004–2007	0.50 (0.46–0.55)	0.52 (0.38–0.71)	0.48 (0.43–0.54)	0.53 (0.45–0.63)
		2012–2015 / 2008–2011	0.74 (0.66–0.83)	0.73 (0.50–1.05)	0.75 (0.64–0.86)	0.91 (0.74–1.12)
Women	Finland	2004–2007 / 2000–2003	0.82 (0.69–0.98)	0.92 (0.63–1.34)	0.86 (0.64–1.17)	0.77 (0.59–1.00)
		2008–2011 / 2004–2007	0.73 (0.59–0.88)	0.56 (0.36–0.85)	0.77 (0.56–1.06)	0.79 (0.57–1.09)
		2012–2015 / 2008–2011	0.80 (0.64–1.00)	0.99 (0.64–1.56)	0.66 (0.46–0.94)	0.95 (0.65–1.38)
	Estonia	2004–2007 / 2000–2003	1.06 (0.80–1.40)	1.25 (0.79–1.97)	0.94 (0.59–1.49)	1.01 (0.60–1.73)
		2008–2011 / 2004–2007	0.71 (0.53–0.95)	0.52 (0.32–0.85)	0.95 (0.60–1.50)	0.66 (0.34–1.28)
		2012–2015 / 2008–2011	0.79 (0.56–1.11)	0.85 (0.48–1.51)	0.86 (0.53–1.39)	0.54 (0.21–1.41)
	Latvia	2004–2007 / 2000–2003	0.85 (0.72–1.01)	1.00 (0.63–1.59)	0.95 (0.75–1.21)	0.71 (0.53–0.95)
		2008–2011 / 2004–2007	0.69 (0.57–0.84)	0.61 (0.36–1.02)	0.72 (0.55–0.93)	0.71 (0.49–1.04)
		2012–2015 / 2008–2011	0.66 (0.52–0.85)	0.82 (0.47–1.43)	0.66 (0.47–0.91)	0.78 (0.45–1.33)
	Lithuania	2004–2007 / 2000–2003	1.23 (1.06–1.43)	1.43 (0.87–2.33)	1.31 (1.06–1.60)	1.18 (0.93–1.51)
		2008–2011 / 2004–2007	0.53 (0.45–0.62)	0.73 (0.48–1.12)	0.50 (0.41–0.62)	0.54 (0.41–0.73)
		2012–2015 / 2008–2011	0.77 (0.63–0.93)	0.47 (0.28–0.79)	0.87 (0.68–1.12)	0.86 (0.58–1.26)

Rate ratios (RR) are adjusted for age and calculated using Poisson regression, RRs measure the changes between two consecutive periods with each preceding period used as the reference category (RR=1); CI, confidence interval.

Supplementary Table 2. Impact of excluding register-based census records on road traffic accident mortality in the 30–74 age group in Latvia, 2000–2015

Sex	Period	Census + registry		Census		Difference in ASMR, %
		ASMR	RR (95% CI)	ASMR	RR (95% CI)	
Men	2000–2003	48.2	-	47.3	-	-1.9
	2004–2007	39.5	0.82 (0.75–0.90)	39.3	0.84 (0.76–0.92)	-0.5
	2008–2011	22.0	0.56 (0.50–0.62)	21.2	0.54 (0.48–0.61)	-3.6
	2012–2015	19.4	0.88 (0.78–1.01)	18.9	0.89 (0.77–1.02)	-2.6
Women	2000–2003	11.3	-	11.0	-	-2.7
	2004–2007	10.2	0.89 (0.76–1.05)	9.7	0.87 (0.73–1.03)	-4.9
	2008–2011	6.8	0.67 (0.56–0.80)	6.6	0.69 (0.57–0.83)	-2.9
	2012–2015	4.4	0.67 (0.53–0.84)	4.4	0.69 (0.54–0.88)	0.0

Rate ratios (RR) are adjusted for age and calculated using Poisson regression with the preceding period as a reference category (RR=1); ASMR, age-standardised mortality rate per 100 000 person years; CI, confidence interval.
